# Patient self-management in primary care patients with mild COPD – protocol of a randomised controlled trial of telephone health coaching

**DOI:** 10.1186/s12890-015-0011-5

**Published:** 2015-02-22

**Authors:** Manbinder S Sidhu, Amanda Daley, Rachel Jordan, Peter A Coventry, Carl Heneghan, Sue Jowett, Sally Singh, Jennifer Marsh, Peymane Adab, Jinu Varghese, David Nunan, Amy Blakemore, Jenny Stevens, Lee Dowson, David Fitzmaurice, Kate Jolly

**Affiliations:** Research Fellow, Primary Care Clinical Sciences, University of Birmingham, Birmingham, UK; Primary Care Clinical Sciences, University of Birmingham, Birmingham, UK; Department of Public Health, Epidemiology and Biostatistics, University of Birmingham, Birmingham, UK; Centre for Primary Care: Institute of Population Health, University of Manchester, Manchester, UK; Nuffield Department of Primary Care Health Sciences, University of Oxford, Oxford, UK; School of Health and Population Sciences, University of Birmingham, Birmingham, UK; Department of Cardiac and Pulmonary Rehabilitation, University Hospitals of Leicester NHS Trust, Leicester, UK; Primary Care Research Network Central England, Telford, UK; Royal Wolverhampton NHS Trust, New Cross Hospital, Wolverhampton Road, Wolverhampton, WV10 0QP UK

**Keywords:** COPD, Self-management, Physical activity, Randomised controlled trial

## Abstract

**Background:**

The prevalence of diagnosed chronic obstructive pulmonary disease (COPD) in the UK is 1.8%, although it is estimated that this represents less than half of the total disease in the population as much remains undiagnosed. Case finding initiatives in primary care will identify people with mild disease and symptoms. The majority of self-management trials have identified patients from secondary care clinics or following a hospital admission for exacerbation of their condition. This trial will recruit a primary care population with mild symptoms of COPD and use telephone health coaching to encourage self-management.

**Methods/Design:**

In this study, using a multi-centred randomised controlled trial (RCT) across at least 70 general practices in England, we plan to establish the effectiveness of nurse-led telephone health coaching to support self-management in primary care for people who report only mild symptoms of their COPD (MRC grade 1 and 2) compared to usual care. The intervention focuses on taking up smoking cessation services, increasing physical activity, medication management and action planning and is underpinned by behavioural change theory. In total, we aim to recruit 556 patients with COPD confirmed by spirometry with follow up at six and 12 months. The primary outcome is health related quality of life using the St Georges Respiratory Questionnaire (SGRQ). Spirometry and BMI are measured at baseline. Secondary outcomes include self-reported health behaviours (smoking and physical activity), physical activity measured by accelerometery (at 12 months), psychological morbidity, self-efficacy and cost-effectiveness of the intervention. Longitudinal qualitative interviews will explore how engaged participants were with the intervention and how embedded behaviour change was in every day practices.

**Discussion:**

This trial will provide robust evidence about the effectiveness of a novel telephone health coaching intervention to promote behaviour change and prevent disease progression in patients with mild symptoms of dyspnoea in primary care.

**Trial registration:**

Current controlled trials ISRCTN06710391.

## Background

### Epidemiology and costs of COPD

Chronic obstructive pulmonary disease (COPD) is a chronic progressive disease with an increasing burden on the NHS and society. The prevalence of diagnosed COPD in the United Kingdom (UK) is 1.8% [1], although it is estimated that this under-represents the burden of disease, [2-4] with the global prevalence estimated as 5-10% [5]. In the UK, COPD accounts for at least 1.4 million consultations with general practitioners (GPs), 1 million in-patient days annually, high health care costs (largely driven by inpatient costs for people with severe disease) [6], and is a leading cause of death [7]. Increasing recognition of the importance of this disease [8,9] culminated in a new National Clinical Outcomes Strategy in 2011 in the UK [10].

### Self-management of COPD

‘Self-management’ has been defined as “the ability of a patient to deal with all that a chronic disease entails, including symptoms, treatment, physical and social consequences and lifestyle changes” [11]. For COPD, the important self-management health behaviours are: smoking cessation, adherence to medication; early recognition of symptoms; prompt access to treatments during an exacerbation (action planning), breathing techniques, bronchial hygiene techniques, exercise, nutritional programmes and stress management [12-14]. The importance of the different elements will vary according to the severity of the condition.

The UK National Clinical Outcomes Strategy recommends early identification of COPD in people [10], thus there is a need for identifying interventions that will maintain function in people with mild symptoms of COPD. With the exception of smoking cessation, few such interventions have been proposed, much less adequately trialled. Clearly smoking cessation is important and while there is some evidence that pharmacological interventions (i.e. varenicline) improve cessation among mild to moderate COPD [15] there is little work on behavioural interventions among this population and people generally prefer non-drug interventions. One effective behavioural intervention for smoking in COPD was led by GPs, which is not a practicable or feasible way to deliver these types of interventions in primary care; it was also tested in China so may well have limited external validity [16]. Based largely on observational evidence [17,18] promotion of physical activity should also be a key component of any intervention. Physical activity declines in patients with COPD, even those with mild disease (GOLD stage 2) and people with mild symptoms (MRC grade 2) [19]. Large-scale observational studies report an association between higher levels of physical activity in people with COPD and reduced hospital admissions and all-cause mortality, even when adjusted for severity [20,21]. A systematic review of five small trials evaluating the effectiveness of physical activity programmes in people with mild/moderate COPD reported improved exercise capacity, but had no effect on dyspnoea or quality of life [22], but other evidence from pulmonary rehabilitation programmes in this group do show improvements of these outcomes [23]. Other important elements for patients with mild dyspnoea are likely to be adherence to medication and action planning at the first signs of a chest infection/exacerbation and exercise.

### Effectiveness of self-management support for COPD

The most recent Cochrane systematic review of self-management interventions [24] (excluding studies on pulmonary rehabilitation) reported that self-management interventions, with significant support from health care workers, delivered to COPD patients in the stable state could significantly reduce hospital admissions compared with usual care (OR 0.60; 95% CI 0.40 to 0.89, 6 studies) and significantly improve health related quality of life (HRQoL) (mean difference in SGRQ −3.51, 95% CI −5.37 to −1.65, 10 studies). This effect was larger than seen in a previous review of limited self-management education alone [25] and approaches the minimally clinically important difference of 4 points [26].

A systematic review of five trials on the effectiveness of action plans only (with only limited education) found that although patients were significantly more likely to recognise exacerbations and initiate treatment, there was no reduction in healthcare utilisation, and the authors concluded that a more significant self-management approach might be needed [27]. A further systematic review of COPD disease management programmes [28] including 10 trials and three before/after studies indicated that such programmes (which often include self-management components) may decrease hospital admissions and improve quality of life, although further exploration of the elements that bring the greatest benefit are needed.

The majority of trials of self-management have identified patients from secondary care clinics or following a hospital admission for exacerbation of their condition. These patients have more severe disease and are not representative of primary care patients, many of whom will have mild COPD.

### Telephone health coaching interventions in COPD

Many self-management interventions involve group-based support or one-to-one consultations; a less expensive delivery option is potentially by telephone. Telephone health coaching is distinct from tele-health, where remote technology is used to monitor disease. Telephone health coaching has been described as: ‘*A method of patient education that guides and prompts a patient to be an active participant in behaviour change. Coaching involves an interactive approach with the patient that helps to identify impediments to behaviour change, and methods of teaching and modelling behaviour that empower the patient to achieve and maintain improved health status. Goal setting and empowerment are important features’* [29]. A rapid review of telephone coaching for long-term conditions reported potential benefits on self-efficacy, health behaviour and health status [30]. However, the evidence for telephone coaching support in COPD is limited. Walters and colleagues undertook an RCT of telephone health mentoring in patients with COPD in Australia and reported improved self-management capacity, lower anxiety, but no effect on health related quality of life [18] and that the telephone intervention was favourably received [31]. A small RCT of a telephone support intervention following hospital discharge increased patient self-efficacy for self-management of their condition [32] and a trial of 172 patients receiving follow-up telephone calls after pulmonary rehabilitation suggested modest additional improvements [33]. A study of a two-week telephone supported intervention in 21 patients with stable but severe COPD [34] significantly increased domiciliary activity levels, exercise capacity, and quality of life scores, although there was no control group.

Although the evidence among COPD patients is limited, physical activity counselling and motivational interviewing by telephone have shown some success in older primary care patients [35] and long-term cancer survivors [36]. An evaluation of a telephone care management service for people with poorly controlled diabetes reported improvement in HbA1c, blood pressure and body mass index [37]. A systematic review of telephone interventions for physical activity in healthy and patient populations reported positive outcomes in 69% of studies [38]. Factors associated with more positive outcomes were intervention duration of at least six months and greater numbers of telephone contacts.

### Need for a trial

There is an absence of research evidence about the effectiveness of self-management interventions in primary care for people who report only mild symptoms of their COPD. This is important, as access to services may be on the basis of symptoms, rather than disease severity and mild symptoms may not mean that airflow obstruction is also mild. Ongoing trials of self-management will not address this need. With the interest in case-finding for COPD in primary care it is important to identify effective interventions, so as to inform care for this patient group. Established self-management programmes for highly symptomatic patients are not necessarily relevant for this group.

The main aim of this phase III trial is to determine whether telephone health coaching to support self-management improves health-related quality of life at 12 months follow-up post-randomisation compared with usual primary care. Additional aims will determine whether the intervention improves self-reported and objective physical activity, smoking and self-management behaviours, psychological morbidity and self-efficacy. We will examine the experience of participants who were offered the supported self-management intervention and assess the cost-effectiveness of the supported self-management intervention.

## Methods

### Design

PSM COPD is a multi-centre RCT of a telephone health coaching intervention to support self-management compared with usual care for people with COPD with mild dyspnoea.

### Study setting

Seventy general practices within England. Additional practices may be added if necessary to meet recruitment targets.

### Study population

Patients are identified as eligible if they are:

- On the practice COPD register, thus have respiratory symptoms.
- Have mild dyspnoea (MRC grades 1 (only breathless on strenuous exercise) or 2 (only get short of breath when hurrying on level ground or up a slight hill)).
- Have a FEV1/FVC < 0.7 after post-bronchodilator spirometry (consistent with current UK guidance [39]) at the baseline assessment. If there is a contraindication to spirometry or the person refuses, a spirometry result within 18 months, may be used, provided the print out can be reviewed and the quality of the test established.
- Aged 18 years or over.

### Exclusion criteria

- Potential participants are excluded if they report their level of dyspnoea to be MRC 3 or greater.
- GPs are asked to exclude participants who, in their opinion, it would be inappropriate for the research team to invite to take part in the trial (e.g. terminal disease, severe psychiatric disorder).

### Patient invitation

**Figure 1:**
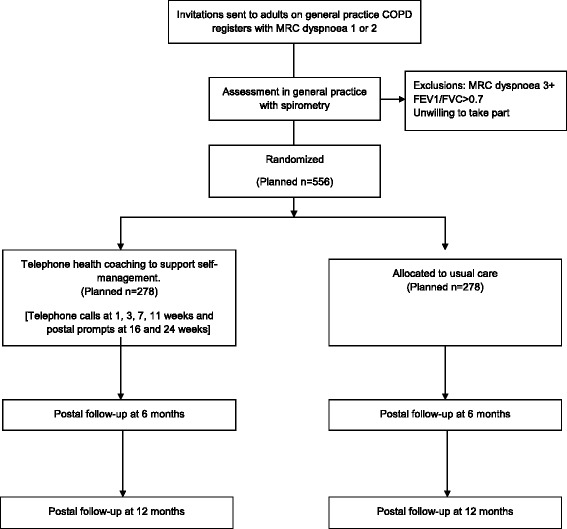
**Study flow diagram.**

Patients identified from COPD registers in general practices receive a letter of invitation on their general practice headed paper, information brochure, patient information leaflet (PIL) and a reply slip including the MRC dyspnoea scale. Patients are asked to return their reply slip with their contact details and MRC dyspnoea level to the research team who contact them to book an assessment at their GP surgery. Participant flow through the study is summarised in Figure 1.

### Baseline assessments

Baseline assessments take place in a room at the patient’s GP practice. Patients attending baseline assessments discuss the trial with the researcher and provide written consent to participate. Permission is also sought to contact participants for future research studies. Post-bronchodilator spirometry is undertaken according to ATS/ERS 2005 guidelines [40] and carried out by research nurses and researchers trained to high standards using a short modified programme modelled on the ARTP Spirometry course. FEV_1_ and FVC are measured using the Easy One spirometer (ndd, Switzerland). Patients are administered 400 micrograms salbutamol through a spacer, and asked to rest for 20 minutes prior to undergoing spirometry. Easy on-PC software is used to read and interpret the spirometry outputs. COPD is confirmed among patients with respiratory symptoms if their FEV1/FVC ratio is less than 0.7. All spirometry results are provided for their GP. Patients’ height is measured to the nearest cm using a practice-based tape measure (wall mounted) or a portable stadiometer (or estimated using arm-span). Weight (to the nearest 0.1 kg) is measured using GP configured scales by research assistants.

During the assessment appointment patients also complete a questionnaire including all the measures collected as outcomes, plus demographic information.

Participants are fitted with a GENEactiv wrist worn accelerometer to be worn for 7 days and returned by post in a pre-paid envelope.

### Allocation to trial arm

When a patient is identified as eligible for the study, and has given written, informed consent to take part, the researcher randomises the patient using a web-based programme hosted by the Primary Care Clinical Trials Unit (PC-CTU), University of Birmingham. Centre specific randomisation lists were produced by a statistician at the trials unit. Participants are individually randomised, stratified by centre. The four recruitment centres are Birmingham and West-Midlands South; Greater Manchester; North West Midlands and Oxfordshire/Gloucestershire. Only the PC-CTU have access to the allocation sequence.

### Usual care

The usual care group receives a standard information leaflet about self-management of COPD. The 13 page leaflet gives a definition of COPD, a detailed description of associated symptoms, how the illness can be managed with the use of inhalers, how to treat exacerbations, and details of other resources (British Lung Foundation, Smokefree-NHS Choices) which the patient might find useful.

### Intervention

The intervention comprises a self-management package of four components: smoking cessation advice; encouragement to become physically active; correct inhaler technique and medication adherence. For those participants with recurrent exacerbations who have an action plan and rescue pack of antibiotics or steroids, there is an exploration of their confidence in identifying an exacerbation early and commencement of medication.

In keeping with best practice the intervention includes education, monitoring and assessment of progress and teaches skills with the aim of increasing self-efficacy [41-43]. We have also taken into account the best evidence for the promotion of physical activity [38,44,45].

The intervention is delivered by telephone by a nurse with one initial 35–60 minute coaching session one week post randomisation followed by three 15–20 minute telephone contacts over a three month period (at approximately weeks 3, 7 and 11) with individually tailored written supportive materials following telephone contacts (e.g. summary of goals agreed, physical activity diary, contact details for local services, information leaflet showing correct inhaler technique). This is followed by standard written prompts/information at 16 and 24 weeks. The first consultation addresses all the elements of the intervention, with subsequent ones addressing physical activity and other elements as appropriate. The intervention components are summarised in Table 1.

**Table 1:** **Summary of intervention components**

**Timing**	**Telephone**	**Postal**
Week 1	Timing of last check of inhaler. If not checked, encourage to get technique checked.	Physical activity booklet
		Physical activity diary
	Smoking behaviours and encouragement to contact smoking cessation service.	Pedometer with instructions
		Smoking booklet (smokers only)
	Current physical activity levels and breathlessness, goal to increase activity and record in diary.	Inhaler technique instruction leaflet
	Discussion of management of exacerbations, do they have an action plan, confidence with use of rescue pack.	
Week 3	Discussion of progress with goals set in previous session and any barriers to achieving goals.	Information on opportunities for physical activity in the locality
	Review of physical activity levels and setting of new goals.	
		Information leaflet: What are SMART goals?
	Discussion of smoking, medication management and action planning as required.	
		SMART goals sheet
Week 7	Discussion of progress with goals set in previous session and any barriers to achieving goals.	SMART goals sheet
	Review of physical activity levels and setting of new goals.	
	Discussion of smoking, medication management and action planning as required.	
Week 11	Discussion of progress with goals set in previous session and any barriers to achieving goals.	SMART goals sheet
	Review of physical activity levels and setting of new goals.	
	Discussion of smoking, medication management and action planning as required.	
Week 16	None	SMART goals sheet
Week 24	None	Information on opportunities for physical activity in the locality
		Leaflet on tips for sustaining physical activity

#### Smoking cessation

The nurse provides information about the health consequences of smoking and sends a booklet with details about the benefits of smoking cessation. The pros and cons for the individual of smoking cessation are discussed. Participants are encouraged to commit to approaching the smoking cessation service and to set a goal to read the smoking cessation literature or contact the service. Any goals are reviewed at subsequent telephone consultations and discrepancy between goals and actions discussed. Participants willing to consider smoking cessation are encouraged to seek social support from family and friends in their quit attempt. Written prompts also provide information about local smoking cessation services in case of relapse or for those not ready to change early on in the intervention period.

#### Physical activity

In terms of promotion of physical activity, the intervention is underpinned by Social Cognitive Theory [46] and aims for participants to achieve the national recommendation for 150 minutes of moderate intensity physical activity each week. The first coaching session centres on uptake of physical activity, reduction of inactivity [47] and focuses on enhancing motivation, self-efficacy for physical activity, overcoming barriers and developing appropriate activity plans and feelings and fears about physical activity, particularly in relation to breathlessness. The final stage of the session involves developing a physical activity goal for the next telephone call. Participants are posted a pedometer to be used as a motivational tool and to assist participants in quantifying the amount of activity they are achieving each day/week. Previous research with other populations has reported pedometers to be effective in increasing the number of steps that individuals take per day [48]. Following the first session a booklet on physical activity and COPD, a diary for recording their physical activity and a reminder of their goals are posted. The follow-up telephone calls focus on goal setting for increasing physical activity and intensity of activity and prevention of relapse back to sedentary behaviour.

#### Medication management

The nurse asks about medication adherence, ascertains confidence with inhaler technique and ascertains when technique was last checked by a health professional. Participants who have not had a recent inhaler check are asked to set a goal to have it checked at a routine practice nurse/GP appointment or by their local pharmacist, and to ask for an annual check. Participants are posted written instructions for using their inhaler/s.

If participants have reported difficulties in remembering to take their medications they are encouraged to self-monitor their behaviour with a written log, to restructure their physical environment to prompt their memory, to consider a medication box or alarm for a reminder, or to seek support from a partner.

Participants are asked about exacerbations and use of antibiotics or steroids for these. Their ability to recognise the symptoms of an exacerbation is checked. People who have been given a prescription to start antibiotics/steroids themselves will be asked about their confidence in starting this medication and asked to describe the last time they had an exacerbation and when and how they made the decision to start their rescue medication. People who are not confident in the use of their action plan are asked to discuss this with their GP or practice nurse at their next routine appointment.

### Intervention fidelity

A sample of telephone consultations are being recorded with the participants consent. These will be assessed according to delivery of the planned components, the use of behavioural change techniques and patient centred approach. The nurses delivering the intervention record the number of contacts and actions taken; these records will be used to assess the proportion of the intervention participants who participated in the intervention, the number of calls per participant and the number who completed all six intervention contacts.

### Outcomes

The primary outcome is health related quality of life using the St Georges Respiratory Questionnaire (SGRQ) [49] which has previously been shown to be responsive in a population with less advanced COPD [50]. The primary endpoint is 12 months from randomisation.

Secondary outcomes: self-reported health behaviours [smoking and physical activity (IPAQ)] [51], self-management activities, psychological morbidity (Hospital Anxiety and Depression Scale) [52], self-efficacy for managing their COPD and undertaking physical activity, MRC dyspnoea scale [53], health care utilisation (primary care consultations for COPD; prescriptions for antibiotics for COPD; hospital admissions for COPD; attendance at smoking cessation service), EuroQoL EQ-5D-5 L [54], physical activity at 1 year measured by accelerometry. Table 2 summarises the outcome measures and timing of assessment.

**Table 2:** **Outcome measures and time of assessment**

	**Baseline**	**6 months**	**12 months**
St George’s Respiratory Questionnaire	X	X	X
Breathlessness: MRC Dyspnoea Scale	X	X	X
Self-management activities	X	X	X
Health related quality of life: EUROQOL EQ-5D-5 L	X	X	X
Psychological status: Hospital Anxiety and Depression Scale	X	X	X
Physical activity measured by GENEactiv accelerometers	X		X
International physical activity Questionnaire (short)	X	X	X
Stanford self-efficacy for COPD and physical activity	X	X	X
Health care utilisation	X	X	X
Post-bronchodilator spirometry	X		
Weight and height	X		
Demographic characteristics	X		
Current medications for lung problems	X		
Co-morbidities	X		

We ask participants randomised to the intervention to complete exercise diary logs that detail the dose and type of physical activity completed during weeks 3, 7, 11 of the intervention. These logs are completed at the end of support calls. At six months all participants are asked about attendance at a smoking cessation service. The research nurses record duration of calls and any adverse events reported by intervention participants.

### Follow up assessments

Outcomes are measured at six months to determine short–term changes to the end of the intervention and 1 year to determine whether change is sustained.

### Statistical justification for sample size

The sample size has been determined to detect a significant difference in the SGRQ at 1 year. To have 80% power to detect a difference of 4 points (which is the minimal clinically significant difference [26] from a baseline total value of 39 [50], with a standard deviation of 15) at the 5% level of significance, 445 evaluable participants are required. We aim to recruit 556 participants, which allows for a 20% loss to follow-up.

This sample size is more than sufficient to detect an increase in physical activity in the intervention group. Assuming the intervention arm reaches 89 minutes of moderate intensity physical activity/week, and the control arm 55 minutes (SD = 70 mins), for 90% power at a 5% significance level, a sample size of 90 patients in each arm is required (in absence of good COPD data, EXERT trial data used) [55]. Accounting for 20% loss-to follow-up, we therefore require a total of 225 patients. To estimate the power for smoking cessation we have taken a smoking prevalence of 45% [56]. Estimates of the likely quit rate are 34% in the intervention arm and 9% in the usual care arm (estimates from Cochrane review of nurse-led interventions for smoking cessation [53]). Our proposed sample size of 278 per group is sufficient to achieve power of 75%, at the 5% significance level.

### Analysis

All data will be analysed by intention to treat. A comparison between groups for the primary outcome measures (health related quality of life) will be made at 1 year to assess the long-term effect of the self-management intervention.

For outcomes measured on a continuous scale (HRQoL, physical activity, EuroQol EQ-5D), analysis of covariance will be used; i.e. a linear regression where the final score is regressed against treatment group and adjusted for baseline score. Model assumptions will be checked and a transformation for Normality used if necessary. Binary outcomes will be analysed using logistic regression and recurrent outcomes (e.g. number of hospital admissions) will be analysed using Poisson regression with an offset term for the length of follow-up. Differences between treatment groups will be summarised with suitable effect estimates (e.g. mean difference, relative rate) with 95% confidence intervals. A 5% statistical significance level will be used. Levels and patterns of missing data will be assessed and summarised, and if appropriate, suitable methods (e.g. regression imputation using baseline data) will be used to evaluate any effect this might have on the conclusions.

We will undertake exploratory subgroup analyses to explore the effects of the intervention in a subgroup who actively engage with the intervention (through increased physical activity, uptake of smoking cessation support or checking of inhaler technique) and those who increase their self-efficacy for COPD self-management. Additionally we will explore effectiveness in subgroups by baseline level of self-efficacy, depression and anxiety scores and HRQoL and participant characteristics (age, sex, ethnicity and number of co-morbidities).

### Economic analysis

#### Within-trial analysis

The health economic analysis will estimate the cost-effectiveness of the self-management intervention compared with usual care. A cost-consequence analysis will initially be reported, describing all the important results relating to costs and consequences in the intervention and control arm over the 1 year trial period. Subsequently, a cost-utility analysis will be undertaken using patient responses to the EuroQoL EQ-5D-5 L questionnaire, to calculate the cost per additional Quality-Adjusted Life Year (QALY) gained over the same period. As there is evidence that the EQ-5D (3-level version) is insensitive to change in pulmonary rehabilitation interventions [57], the more recently developed 5 level version will be used in this evaluation. QALYs will be calculated using data on mortality and EQ-5D-5 L responses at baseline, six and 12 months.

The base-case cost analysis will adopt an NHS perspective and will include costs of the intervention, usual care, and condition-specific health care utilisation (primary and secondary care) including emergency admissions for exacerbations and medication costs. Information on condition-specific resource use, including standard care will be collected from using patient questionnaires. Data on additional costs of the intervention including practice nurse time, telephone support, web-site support, written materials and patient prompts, exercise, additional GP consultation time and smoking cessation will be collected alongside the trial. Data will also be collected on set up and implementation costs such as staff training workshops and implementing quality control measures. Unit costs will be obtained from standard sources and health care providers, including NHS reference costs. Information will also be collected on time off work related to COPD and will enable analysis for a broader societal cost perspective.

The robustness of the base-case results will be explored using sensitivity analysis. This will explore uncertainties in the trial based data itself and the methods employed to collect and analyse the data. Uncertainty will be explored through the use of cost-effectiveness acceptability curves (CEACs), which will estimate the probability that self-management is cost-effective at different cost per QALY thresholds.

#### Model-based analysis

If the self-management intervention demonstrates effectiveness within the trial, a Markov model-based analysis will also be undertaken to determine the cost per QALY gained beyond the trial period. The model structure will be informed by a number of complementary methods. It will build on modelling work undertaken for two funded COPD studies on case finding and self-management for severe to moderate COPD which will include a review of existing decision models in COPD, and incorporate patient pathways identified within this research framework. The model will be populated using the information on the costs and outcomes demonstrated within the trial and extrapolated to estimate the long-term costs and effects.

The robustness of the model results will be explored using sensitivity analysis. Deterministic sensitivity analysis will vary point estimates for all important input parameters within a range of possible values (confidence intervals). Probability distributions around point estimates will be used to explore the uncertainty in the confidence to be placed on the results of the economic analysis, using probabilistic sensitivity analysis to estimate CEACs. Differences in cost and outcomes associated with patient characteristics such as sex, age, smoking status and existing co-morbidities may be explored through subgroup analysis, dependent on data availability.

### Qualitative process evaluation

Our primary research question is to explore the way in which patients with mild COPD understand and respond to a telephone coaching, self-management intervention. We will explore the acceptability and feasibility of the intervention and investigate how and why patients use or discard opportunities for self-care over a 12 month period. Finally, we will explore if self-care behaviours promoted by the intervention become embedded within day-to-day routines.

We will undertake individual interviews with participants using a longitudinal study design over the course of the trial. We will select up to 20 participants who have received the intervention, and up to 10 participants from the control group. Interviews will take place approximately six and 12 months post randomisation. Interviews will continue until saturation is reached [58]. Participants will be purposively sampled to ensure there is maximum variation in the sample along the following key characteristics: HRQoL, geography, gender and age.

Longitudinal approaches have been used to uncover multiple perspectives about disease progression and care provision among patients with end-stage COPD [59], but little is known about changing perspectives among patients with mild to moderate disease who are engaged in self-care. A longitudinal qualitative research design makes it possible to capture any changes in self-management trajectories within the context of the experience of living with and managing a chronic condition such as COPD.

### Regulatory issues

#### Ethics approval

The study has received ethical and research governance approval from the National Research Ethics Service (Reference 13/WM/0206). The study will be conducted in accordance with the recommendations for physicians involved in research on human subjects adopted by the 18th World Medical Assembly, Helsinki 1964 and later revisions.

#### Indemnity

The University of Birmingham holds the relevant insurance policy for this study.

#### Sponsor

The University of Birmingham will act as the main sponsor for this study.

### Funding

National Institute for Health Research School for Primary Care Research (SPCR Project 174).

### Publication policy

We will present the results to the TSC prior to publication. The investigators will be involved in reviewing drafts of the manuscripts, abstracts, press releases and any other publication arising from the study. Members of the TSC will be listed and their contribution acknowledged, as will the funding source (NIHR School for Primary Care Research). Authorship will be determined in accordance with the ICMJE guidelines and other contributors will be acknowledged.

### Patient advisory group

A patient advisory group (PAG) has been set up, chaired by Mr Michael Darby. This group is funded to meet at quarterly intervals or according to need, and will advise on the design, conduct, analysis and dissemination of the study. The PAG will discuss issues raised by the CI and report comment back to investigators.

### Data management

All data will be stored on a password-protected web-enabled database designed and hosted by the PC-CTRU. Paper-based information will be held in locked filing cabinets in the study office.

### Trial steering committee

The Trial Steering Committee (TSC) has been convened to provide overall supervision of the trial and ensure it is in accordance with the principles of good clinical practice and the relevant regulations. The TSC agreed the trial protocol and will agree any protocol amendments. The TSC also provide advice to the investigators on all aspects of the trial. The TSC is chaired by Dr Hilary Pinnock (University of Edinburgh). Dr Rupert Jones (Plymouth University), Dr Jill Mollison (University of Oxford) and Mrs Maireade Bird (patient representative) are members.

### Data monitoring committee

We did not consider that a separate data monitoring and ethics committee would have been useful, as this is an unblinded study with no substantial risk and no early termination rules. The final decision was made by the TSC, who agreed to incorporate the functions of a DMC into their terms of reference.

## Discussion

This trial investigates the effectiveness and cost effectiveness of a nurse-led telephone health coaching intervention to support self-management for patients living with mild symptoms of COPD.

There are many strengths of this study including: a multi-centre study design incorporating a large sample of GP practices representative of the general UK population; it has a pragmatic design to accommodate real life patient practices; spirometry is undertaken using trained staff and quality assured; the intervention is underpinned by behaviour change theory; there is an embedded longitudinal qualitative study; and a full cost effectiveness analyses. As a result, this trial will inform practice across the UK and contribute to the current effort to support people living with COPD.

Nevertheless, a multifaceted trial of this size brings a number of challenges. First, we are approaching a number of practices and identifying thousands of patients. Second, ensuring that we identify suitable patients (meeting our inclusion criteria) from GP databases can create difficulties, as the accuracy of the diagnosis of COPD varies for patients on GP COPD registers. Third, patients who have relatively mild symptoms from the COPD may be less likely to respond to our invitation. Even with relatively mild dyspnoea, potential participants will suffer from exacerbations and chest infections which delay their recruitment and requires some flexibility in the delivery of the intervention. Finally, co-ordinating baseline assessment with patients and GP practices over four main sites is challenging and will require considerable resources to fulfil our recruitment target.

This trial will contribute robust evidence about the effectiveness of a novel telephone health coaching intervention to improve outcomes and delay disease progression among people with mild COPD. The service might be a template for delivery of a flexible intervention that can reach large numbers of an under recognised population and as such it has capacity to contribute significantly to improving public health.

## Abbreviations

ARTP: Association for Respiratory Technology & Physiology
ATS: American Thoracic Society
CEAC: Cost-effectiveness acceptability curves
COPD: Chronic obstructive pulmonary disease
ERS: European Respiratory Society
GP: General Practitioner
HRQoL: Health related quality of life
IPAQ: International physical activity questionnaire
RCT: Randomised controlled trial
NIHR: National Institute for Health Research
QALY: Quality adjusted life year
SGRQ: St George’s Respiratory Questionnaire
UK: United Kingdom
